# Supplementary material for: “The future depends on what we do in the present” - Development positions of EU countries by levels of sustainable development and living standards
Source: PLoS One. 2025 Jul 31;20(7):e0326739. doi: 10.1371/journal.pone.0326739 (PMC12312900; doi:10.1371/journal.pone.0326739)
Supplement: Attachment — Submitted filename: Review Comments.docx [file pone.0326739.s001.docx]

**Review Comments**

The following study highlights the position of European Union (EU) countries and changes in terms of sustainable development and the standard of living of inhabitants covering period from 2015-2021. The study employed modified positional technique for order of preference by similarity to the ideal solution (MP-TOPSIS) which seems an appropriate methodology. Although, the topics of sustainable development and standard of living have been discussed extensively in the past in the context of European Economies; however, the authors have tried to investigate it from a new lens. The paper adds some value to the existing literature, however, it has following discrepancies which needs to be addressed in order for paper to be published in “Plos One”.

**Introduction:**

- The importance of the study has not been properly highlighted as the authors have tried to emphasize its importance in the following paragraph “Our study fills in the 161 research gap in assessing the level of sustainable development in connection with the standard 162 of living. We presented a comprehensive approach to the study of these two complex issues 163 and presented an application to the study of the situation of EU countries.”. However, these are more generalized statements and does not clearly highlight the purpose of the research. Kindly revisit the paragraph for the importance of the study and include the true importance of the study and its practical implications also. In the paragraph, kindly also mention that why you pursued the methodology you are using and compare it with the past literature who has used methodology under similar circumstances.
- The last paragraph of the introduction lacks a proper ending. It should include a statement like “Rest of the paper is organized as follows……...”.

**Literature review**

- The literature review is thorough; however, the referenced material is mainly before 2020. Kindly include some more studies starting from 2023.
- Moreover, literature review seemed to be written in hurry and contains number of grammatical mistakes. There are also sentences which does not make any sense. I have highlighted some of the mistakes but I request the authors to kindly revisit the whole literature review section and remove any grammatical mistakes or otherwise from the paragraphs. Rewrite the entire sentences where appropriate. Here are some of the example sentences to be corrected.

Line 203:

Change the sentence and write in more academic way: They 203 proposed a method for calculating an integrated development index for EU member states 204 during 2016-2017, with scenario forecasting up to 2020.

Line 230:

Correct and rewrite:

Ukraine through the prism of the experience of developed countries, including the EU.

Line 252:

252 under another SDG presented by Łakomy-Zinowik (2022) focusing.” What does under another SDG means. Kindly correct and rewrite.

**Methods and Data:**

- The methods and data section mainly discusses the methodology section and completely misses data section. Kindly first discuss the data and also provide a proper explanation of why the data and the period covering it was chosen. Which EU economies were not included and what was the reason for that. The inclusion of the economy follows what criteria and other such data related discussion.
- The methods and data section have been properly written and explained.

Line 460-461:

- Kindly correct the equation 20 as in logit the “it” should be subscript. Besides that, correct line 461 also. What does “or 2 for standard of living” means. Kindly correct.

Line 464-465:

- Kindly provide a proper reference for the data obtained. Include it as a footnote. Provide whether you gained it from website or a report?

**Results**

Figure 4:

The note is incorrectly written. What does arrows indicate. “Note: In the figure arrows the five largest and five smallest changes in the European Union's countries.”

Kindly also correct “level” to “Level” in the figure.

Provide a proper reference to the figure.

- In the following paragraph “Figure 4 shows the development position of European Union countries in 2015 and 2021. Four main development positions of countries have been identified: strong effective position (10 countries in 2015 and 12 countries in 2021); preemptive position (2 countries in 2015 and 2022); moderate position (7 countries in 2015 and 8 countries in 2021); and weak position (8 countries in 2015 and 5 countries in 2021). Ten countries retained their strong effective position from 2015 to 2021, but in 2021 this group was joined by countries such as Portugal and Slovakia.” You mention preemptive position for 2022 which does not seem to exist in figure 4. Kindly comment
- In the following paragraph “When analysing the data, it was observed that most countries shifted their positions on the sustainable development matrix, indicating a positive development trend. However, there were some exceptions e.g. Austria experienced a decline in its position. This stands out from the overall trend. Additionally, while only Austria shifted itself in the coordinate system, it is not the sole country that deteriorated in terms of sustainable development. The Czech Republic also experienced a slight decline, although it didn't change its position on the sustainable development matrix. This raises questions for further research about what caused these countries and their residents to experience a decline in sustainable development over seven years. Comparing these two cases with the results of other countries can provide valuable insights for remedial actions. However, it's challenging at this stage to conclude the causes of these declines.” Why these relationships exist?
- In the “Results” section there are very few results which have been compared to the existing or past literature. Kindly discuss some key results in the light of the existing literature by providing a proper reference to them.

**Conclusion**

- The opening of the conclusion section indicates that the study investigates the relationship between “sustainable development and the standard of living within the European Union”. However, the main purpose of the study seems to be “changes in terms of sustainable development and the standard of living of inhabitants”. Kindly change the opening of the conclusion section in a way that it highlights 1. Why you conducted the research 2. What methodology you employed 3. The key findings 4. And its practical implications in the paragraph form.
- Kindly change the bullet points to paragraph form in the conclusion section.

**References:**

- Kindly follow the proper guidelines of the journal in references. The references are not uniform as some references contains DOI while it is missing from others. Moreover, the references of the books are not in the proper format.
